# “Failure To Launch”: Development of a Reproductive Organ Linked to Symbiotic Bacteria

**DOI:** 10.1128/mbio.02131-22

**Published:** 2023-01-19

**Authors:** Sarah J. McAnulty, Allison H. Kerwin, Eric Koch, Barrett Nuttall, Andrea M. Suria, Andrew J. Collins, Tyler R. Schleicher, Bethany A. Rader, Spencer V. Nyholm

**Affiliations:** a Department of Molecular and Cell Biology, University of Connecticut, Storrs, Connecticut, USA; b Kewalo Marine Laboratory, Pacific Biosciences Research Center, University of Hawaii at Manoa, Honolulu, Hawaii, USA; University of Hawaii at Manoa

**Keywords:** *Euprymna*, symbiosis, *Verrucomicrobia*, bacteria, development, squid, *Alphaproteobacteria*, *Euprymna scolopes*, accessory nidamental gland, developmental biology, marine bacteria

## Abstract

Developmental processes in animals are influenced by colonization and/or signaling from microbial symbionts. Here, we show that bacteria from the environment are linked to development of a symbiotic organ that houses a bacterial consortium in female Hawaiian bobtail squid, *Euprymna scolopes*. In addition to the well-characterized light organ association with the bioluminescent bacterium Vibrio fischeri, female *E. scolopes* house a simple bacterial community in a reproductive organ, the accessory nidamental gland (ANG). In order to understand the influences of bacteria on ANG development, squid were raised in the laboratory under conditions where exposure to environmental microorganisms was experimentally manipulated. Under conditions where hosts were exposed to depleted environmental bacteria, ANGs were completely absent or stunted, a result independent of the presence of the light organ symbiont V. fischeri. When squid were raised in the laboratory with substrate from the host’s natural environment containing the native microbiota, normal ANG development was observed, and the bacterial communities were similar to wild-caught animals. Analysis of the bacterial communities from ANGs and substrates of wild-caught and laboratory-raised animals suggests that certain bacterial groups, namely, the *Verrucomicrobia*, are linked to ANG development. The ANG community composition was also experimentally manipulated. Squid raised with natural substrate supplemented with a specific ANG bacterial strain, *Leisingera* sp. JC1, had high proportions of this strain in the ANG, suggesting that once ANG development is initiated, specific strains can be introduced and subsequently colonize the organ. Overall, these data suggest that environmental bacteria are required for development of the ANG in *E. scolopes*.

## INTRODUCTION

Microorganisms have had a profound effect on the evolution of eukaryotes. Interactions between hosts and microbes are often beneficial, for example aiding in host nutrition (1–3) or defense against pathogens and predators (4, 5). Many host developmental processes are also influenced by microbiota, including the vertebrate digestive (6) and immune systems (7), and the light organ of the Hawaiian bobtail squid (8). In some organisms, symbionts are required to transition between life stages. In the upside-down jellyfish, *Cassiopea xamachana*, symbiosis onset with *Symbiodinium microadriaticum* is required for metamorphosis (9–11), and in other marine invertebrates, a microbial cue is required for the transition from the pelagic larval stage to the sessile adult stage (9, 12–17). In nonanimal systems, microbes can also change the course of host development (e.g., transition from vegetative mycelium to the mushroom fruiting body in *Agaricus bisporus* (18), or the formation of root nodules in leguminous plants (19)).

Many animal and plant hosts have evolved specialized organs or tissues that house these bacterial symbionts (20) (e.g., digestive tract of mammals (21), light organs of squids and fishes (22, 23), the trophosome of tubeworms (24), bacteriocytes of insects (25), or root nodules of leguminous plants (26)). By controlling the microenvironment of these niches, the host can often select for specific symbionts from the environment (8) and regulate the rate at which these bacterial symbionts grow (27–30). Hosts can also utilize bacterial products, and/or alter the distribution of the symbionts within or outside the host (24). In turn, bacterial colonization can directly influence the development of organ systems in many hosts (21–26). However, in all cases to date, even in the absence of a microbiota (i.e., germfree or axenic animals) the overall structure of an organ will develop, although its function and specific composition may be compromised. Here we show that under certain rearing conditions with an altered environmental microbiome, a symbiotic organ completely fails to develop.

**FIG 1:**
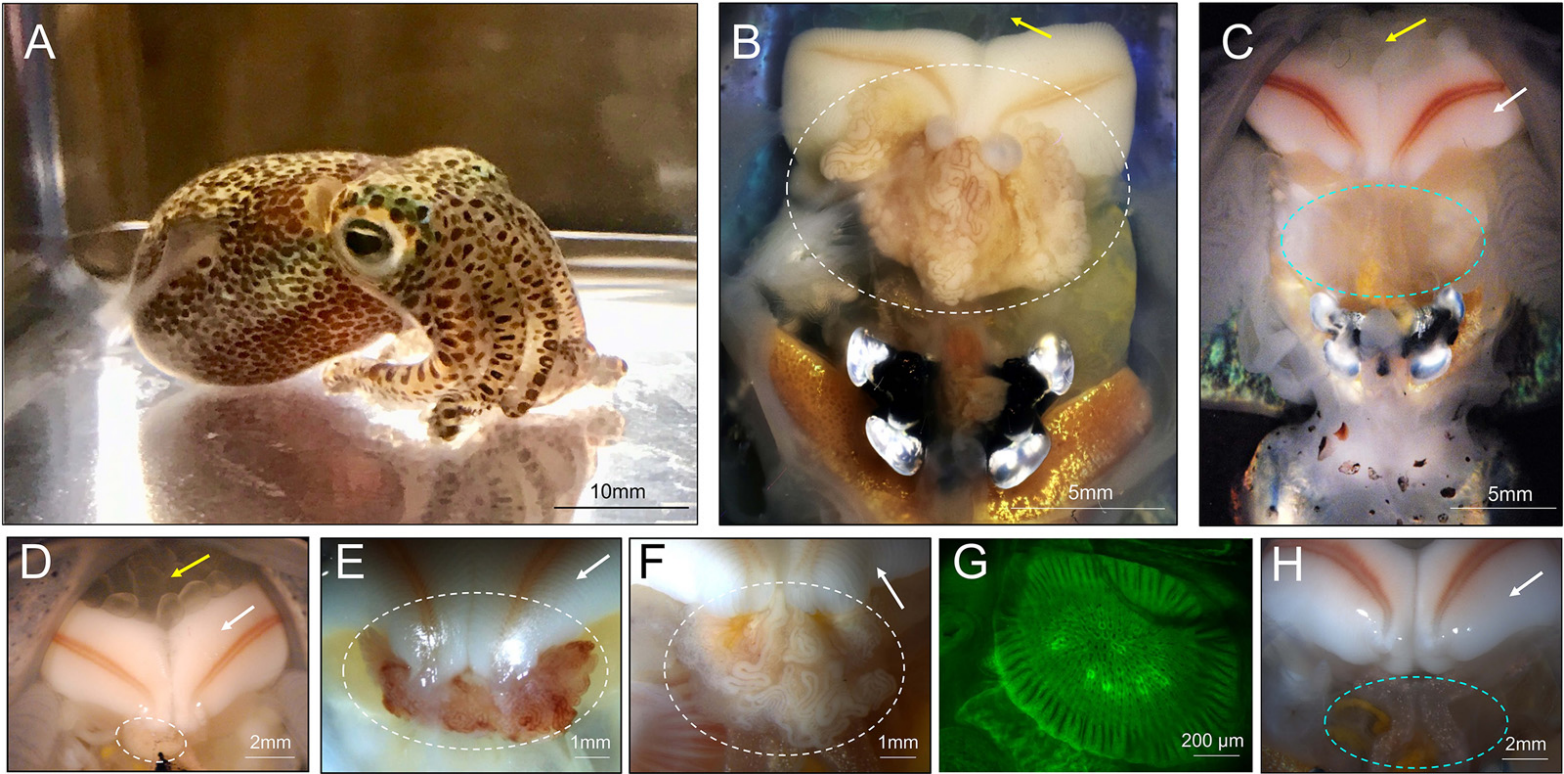
Female Hawaiian bobtail squid develop accessory nidamental glands in response to environmental substrate. (A) Adult Hawaiian bobtail squid. (B) Ventral dissection shows the accessory nidamental gland (ANG, white circle) of a sexually mature wild-caught animal (image modified from Kerwin et al., 2021 [40]). (C) Ventral dissection of a sexually mature lab-raised (lab sand, see Fig. 2) squid, showing where an ANG failed to develop (blue circle). Tissue shown within circle is part of the digestive gland, dorsal to where the ANG would normally be located. (D–H) Representative images depicting the ANGs of animals raised in various conditions, including, (D) lab sand (see Fig. 2) with a stunted ANG (white circle), (E) an ANG from a Wisconsin-raised animal (white circle), (F) an ANG from a Connecticut-raised animal on wet-collected sand (white circle), (G) a confocal micrograph of a nascent ANG from a Connecticut-raised animal on autoclaved wet-collected sand, and (H) an animal raised on dry-collected Hawaiian sand, ANG is absent (blue circle). White arrows indicate fully formed nidamental glands (C–F, H), yellow arrows indicate eggs in mantle cavity (B–D).

The Hawaiian bobtail squid, *Euprymna scolopes*, is a model organism for the study of host-microbe interactions and has been studied primarily for its symbiotic light organ association with the bioluminescent bacterium, Vibrio fischeri (8, 31, 32). Female *E. scolopes* also have a symbiotic organ in their reproductive system, the accessory nidamental gland (ANG), which houses a bacterial consortium within epithelium-lined tubules (Fig. 1) (33). Bacteria from this organ are incorporated into the egg jelly coat (34), where they prevent fungal fouling of the eggs (35). The bacterial consortium of the ANG from *E. scolopes* is dominated by *Alphaproteobacteria* and *Verrucomicrobia*, while *Flavobacteriia* and *Gammaproteobacteria* are also present (33, 34, 36). Some members of the community have been isolated (35, 37–39) and many of these strains have antimicrobial activity against both fungi and/or other bacteria *in vitro* (35, 38, 39). The ANG symbiosis is likely environmentally transmitted, with many of the ANG bacterial species found in the sediment from the host’s environment (34). During development, the host also has a nascent ANG that is poised to recruit environmental bacteria (40). Because of the benthic nature of *E. scolopes* and its behavior of burying in sand during its quiescent diurnal period (41), these environmental bacteria are likely to come into direct contact with the host during all stages of development and during a proposed developmental colonization window when the nascent ANG is covered in ciliated invaginations and ready for colonization (40).

In this study, we raised bobtail squid and examined the effects of different laboratory conditions on ANG development. We demonstrate that under specific rearing conditions, an environmental bacterial community was required for the formation and colonization of the mature organ. Without the presence of this bacterial community, the ANG failed to develop in adult squid.

## RESULTS

### Raising animals in laboratory-maintained substrate did not induce full ANG development.

To examine the development of the ANG under laboratory conditions, female bobtail squid were raised from hatching to adulthood in filter-sterilized artificial seawater and autoclaved substrate (sand), then subsequently transferred to tanks that had previously contained wild-caught squid (n = 18) at the University of Connecticut (CT-raised). Most of the sand in the transfer tanks had been in the lab for at least a year prior to these experiments, although some sand from Maunalua Bay, HI, had been added sporadically.

**FIG 2:**
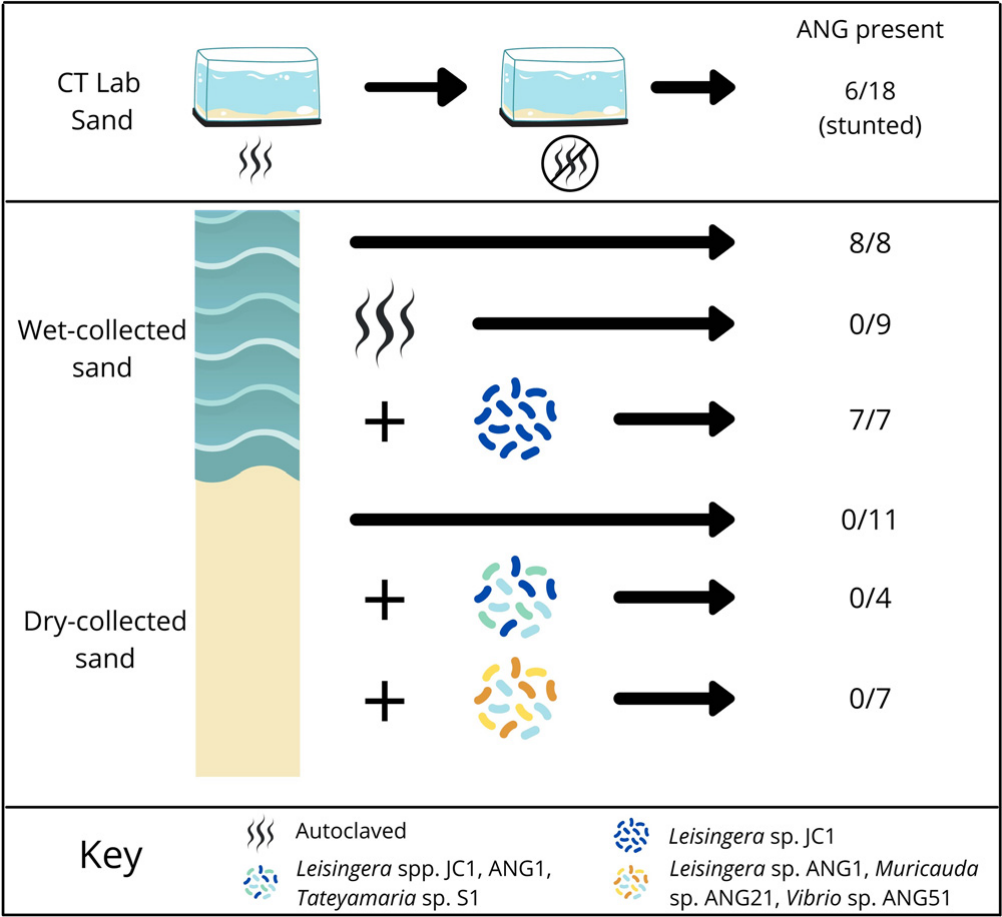
Experimental design and effects of substrate raising conditions on ANG development. Squid raised in Connecticut under “lab sand” conditions: Hatchlings were first raised with autoclaved sand and filter-sterilized artificial seawater until they were approximately 8–10 mm mantle length. They were then transferred to tanks that had previously housed wild-caught squid on unautoclaved sand and raised to sexual maturity. For the “wet-collected conditions,” squid were raised on sand obtained from below the low-tide mark in Maunalua Bay, Oahu, HI. Squid were also raised on wet-collected sand that had been autoclaved prior to rearing. In separate experiments, *Leisingera* sp. JC1 was added to tanks for squid which were also raised on wet-collected sand. For “dry-collected conditions,” squid were raised on substrate collected from above the high-tide mark (see methods), and specific ANG isolates were added to some conditions as shown. Numbers on the right indicate the number of ANGs that formed in adult animals out of the total number of female animals from that condition.

Sixty-seven percent of these squid (n = 18) did not develop any visible ANGs (Fig. 1C), while the remainder (33%) developed ANGs that were asymmetrical and less pigmented than those from wild-caught squid (Fig. 1D, Fig. 2A). The ANGs that developed also appeared stunted in comparison to wild-caught squid, as can be seen from the relative sizes of ANGs and nidamental glands (the secretory organ that produces the jelly coat, compare Fig. 1D and 1B). Animals that lacked ANGs had fully developed nidamental glands and contained eggs, indicating that they were sexually mature (Fig. 1C, arrows).

A separate cohort of animals was independently raised at the University of Wisconsin in artificial seawater on sand originating from Hawaii (here referred to as WI-raised). WI-raised squid consistently developed ANGs, which tended to be symmetrical and more pigmented than ANGs from CT-raised animals maintained on lab sand (Fig. 1E). However, the ANGs from WI-raised squid generally appeared smaller than ANGs from wild-caught animals (Fig. 1E).

**FIG 3:**
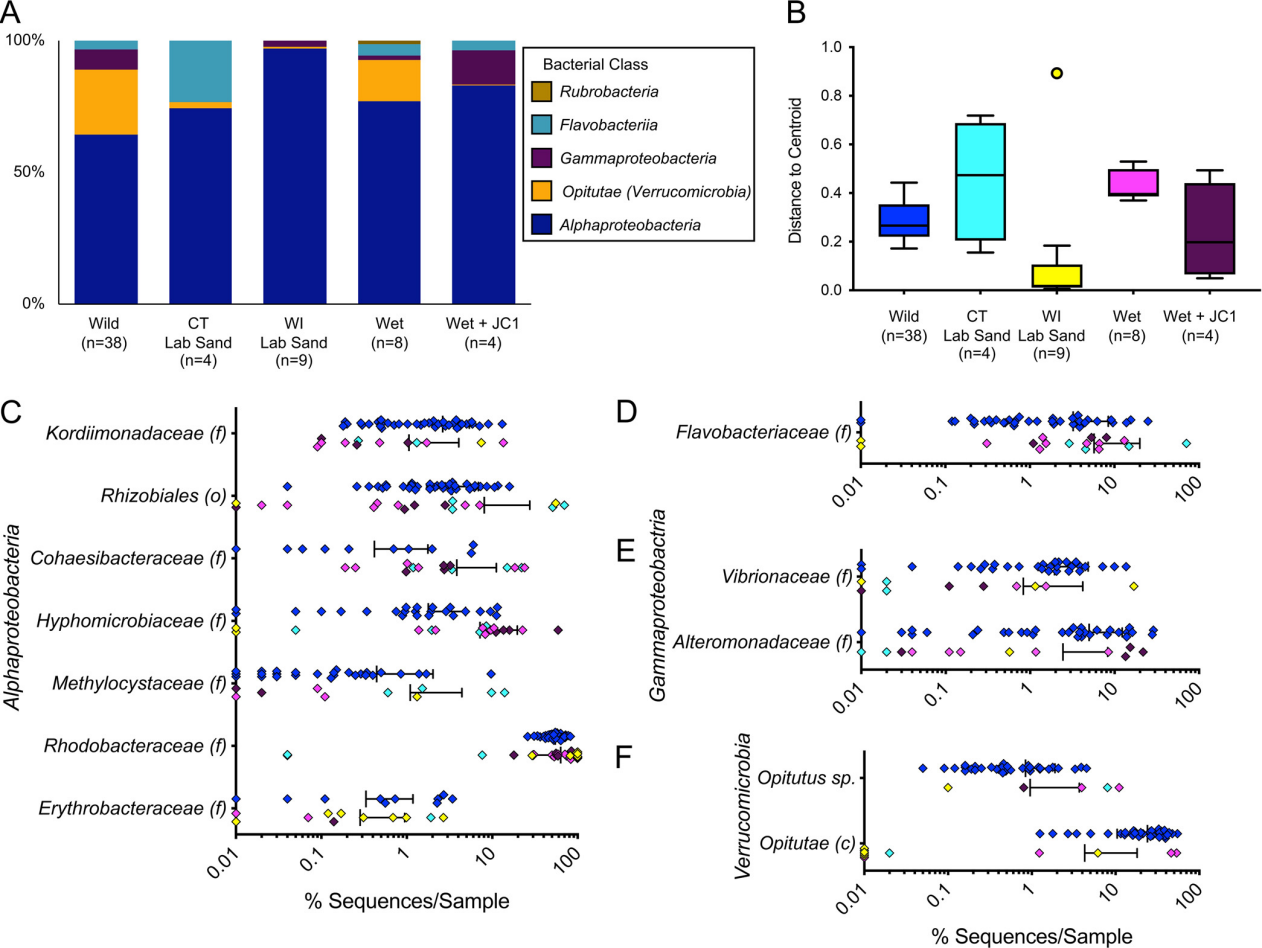
Taxonomic diversity of laboratory-raised *E. scolopes* ANG communities (class level: A, finer levels: C–F) shows that the ANG communities of raised animals were dominated by *Alphaproteobacteria* (A, C), but almost completely lacked *Opitutae* (class of *Verrucomicrobia*) unless animals were raised on wet-collected sand (A, F). *Flavobacteriia* were only found in ANGs from CT-raised animals (D). Raised animals showed high levels of variability for all taxa (C–F), also evident from a plot of the average distance from the beta diversity centroid for each condition (B). Finer level plots (C–F) are shown on a log scale and include samples which contained these taxa at >0.1% abundance (c: class, f: family, o: order). Colors of C–F are the same as those in B. “Wet” indicates wet-collected sand throughout. “Lab sand” samples are from stunted ANGs from raising experiments not conducted on wet-collected sand (Fig. 2). Bars in C–F indicate mean +/− standard deviation.

Bacterial community profiles for ANGs were developed using the V4 region of the 16S rRNA gene as previously described (34, 36). WI-raised ANGs (n = 9) were almost exclusively dominated by *Rhodobacteraceae* (*Alphaproteobacteria*) averaging 89.4 ± 23.3% (Fig. 3A). In contrast, the few stunted ANGs from CT-raised animals on lab sand (n = 4) contained a more diverse bacterial consortium that was dominated by *Alphaproteobacteria* (averaging 20.5 ± 35.9% *Rhodobacteraceae*, and 32.0 ± 33.8% *Rhizobiales*), but also contained a large proportion of *Flavobacteriia*, averaging 23.4 ± 32.3% (Fig. 3A).

### Raising animals in substrate containing the native microbiota induced ANG development.

Previous work found ANG bacterial community operational taxonomic units (OTUs) were present in sediment samples from the host’s environment where wild-caught squid were collected (34). To test whether ANGs would develop in the presence of a microbial community reflective of the host’s natural environment, squid were raised on sand that had been collected from below the low tide zone from Maunalua Bay, Oahu, HI (wet-collected sand; see methods, Fig. 2). Sand was kept wet from the time of collection through being added to the raising tanks. All squid raised under these conditions developed symmetrical ANGs with fully developed tubules (n = 8, Fig. 1F). ANG bacterial communities from animals raised on wet-collected sand most strongly resembled the wild-caught ANG community and consisted of 59.8 ± 16.5% *Rhodobacteraceae*, 4.5 ± 4.3% *Flavobacteriia*, and 15.7 ± 7.3% *Verrucomicrobia* (Fig. 3A).

To examine whether living microorganisms and/or a non-living component of wet-collected sand initiates ANG development, the wet-collected sand was first autoclaved and squid were then raised on this sand with filter-sterilized seawater from hatching to adulthood. None of these squid developed an ANG (n = 9, Fig. 2). However, immature squid from this treatment did contain an ANG primordium that is involved with bacterial recruitment (40) (n = 8, Fig. 1G), demonstrating that in the absence of a viable environmental signal, ANG development is arrested, and the gland is lost by sexual maturity.

### Raising animals in substrate containing native microbiota allowed for colonization by specific ANG strains.

**FIG 4:**
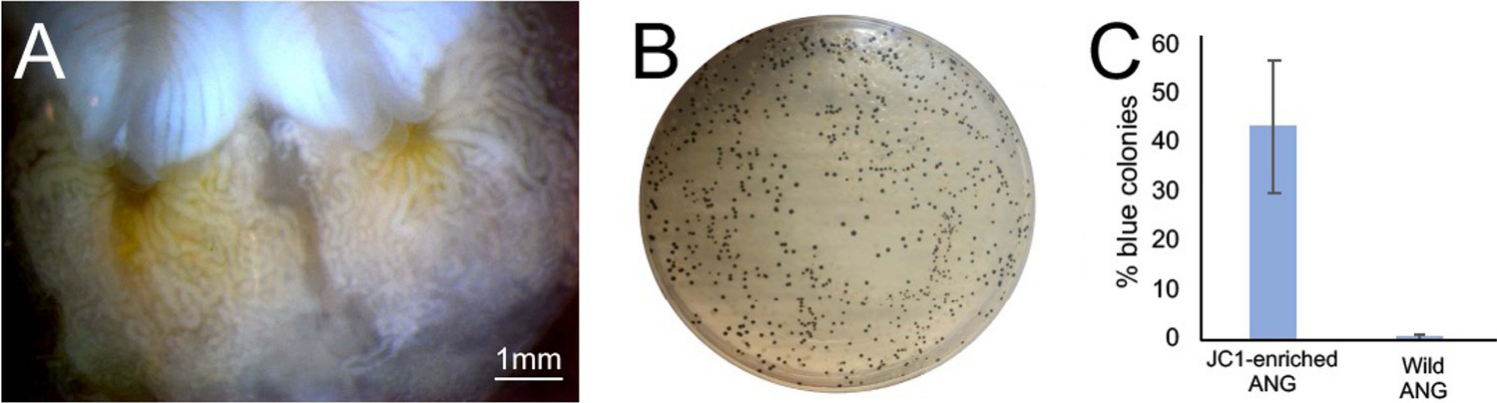
A specific member of the ANG community, *Leisingera* sp. JC1, was incorporated into ANG tubules. (A) A representative image of the ANG of a squid raised on wet-collected Hawaiian sand with the addition of strain *Leisingera* sp. JC1 to the tank water. (B) Representative plate of the cultivable ANG community with blue colonies representing *Leisingera* sp. JC1. (C) On average, 43% +/− 13.5% of colonies plated from ANGs raised on wet-collected sand + JC1 (n = 5) were blue, compared to an average of 0.5% +/− 0.5% of colonies plated from wild-caught ANG homogenate (n = 5). Bars in C indicate mean +/− standard deviation.

In order to determine whether specific bacterial isolates can be incorporated into the ANG consortium, *Leisingera* sp. JC1 was added to tank water of squid raised on wet-collected sand. *Leisingera* sp. JC1 colonies are a distinctive blue-black color when grown in culture due to production of the compound indigoidine and are a rare member of the overall ANG community (38). All squid raised under these conditions developed ANGs (n = 7, Fig. 2, Fig. 4A), and 5 squid (of 6 ANGs sampled) incorporated this strain into the organ (Fig. 4; n = 6). An average of 43 ± 13.5% of all cultured colonies were dark blue (n = 5 ANGs), suggesting that they were *Leisingera* sp. JC1 (Fig. 4B and C). Blue colonies are found at very low abundance in plated ANGs from wild-caught animals, (averaging 0.5 ± 0.5%, n = 5 ANGs, Fig. 4C). Colony PCR of a subset of these blue colonies confirmed the presence of the genes associated with indigoidine production, *igiC*, *igiD*, and *igiR*. The bacterial community of the ANG also shifted in composition compared to animals raised just on wet-collected sand. The relative abundance of *Verrucomicrobia* was only 0.2 ± 0.4% in these ANGs, a shift balanced by a corresponding increase in *Alphaproteobacteria* and *Alteromonadaceae* (Fig. 3A, C, and E).

### Raising animals on dry-collected substrate did not induce full ANG development, even with the addition of ANG isolates.

We next tested whether squid raised on sand collected from above the high tide zone (dry-collected sand) would develop mature ANGs. This dry-collected sand has similar physical properties to the wet-collected sand described above but did not have the same microbial community as the wet-collected sand due to desiccation and sun exposure (see substrate analysis below). To understand whether specific bacterial members of the ANG community may trigger complete organ development, squid were also raised on dry-collected sand, but with the addition of cultured ANG isolates (Fig. 2). In one trial, three *Rhodobacteraceae* strains were added (*Leisingera* spp. JC1 and ANG1, and *Tateyamaria* sp. S1, Table S1). *Rhodobacteraceae* (members of the *Alphaproteobacteria*) are the most abundant bacterial group in the ANGs of *E. scolopes* (34). Squid were also raised on dry-collected sand with the addition of a more taxonomically diverse set of strains from the ANG community (*Leisingera* sp. ANG-DT; *Vibrio* sp. ANG51; and *Muricauda* sp. ANG21, Table S1). All squid raised on dry-collected sand lacked visible ANGs at sexual maturity. No ANG development was observed under these sets of conditions (Fig. 2): no bacteria added (*n* = 11, Fig. 1H), three Rhodobacteraceae strains added (*n* = 4), or taxonomically diverse strains added (*n* = 7). As none of these conditions resulted in ANG development, no ANGs were available for bacterial community profiling.

10.1128/mbio.02131-22.1 TABLE S1 Bacterial strains used in this study. Download Table S1, TIF file, 1 MB. Copyright © 2023 McAnulty et al. 2023 McAnulty et al. https://creativecommons.org/licenses/by/4.0/ This content is distributed under the terms of the Creative Commons Attribution 4.0 International license.

Overall, the results from all raising experiments suggest that specific, but not yet identified, bacterial members of the substrate community induce initial tubule formation and subsequent development of the mature ANG. After full development is induced, the organ can be colonized by specific ANG isolates during establishment of the association, including a strain that on its own was not able to induce ANG development (*Leisingera* sp., JC1, Fig. 2 and 4).

### ANG development was independent of light organ colonization.

To determine whether the presence of Vibrio fischeri and corresponding light organ colonization would impact ANG development, V. fischeri was added to the tank water of a subset of hatchlings from some treatments (both wet-collected and dry-collected sand) while control aposymbiotic animals lacked V. fischeri. The presence of V. fischeri had no impact on ANG development (Table S2).

10.1128/mbio.02131-22.2 TABLE S2 The effect of light organ colonization on ANG development. Download Table S2, TIF file, 2.2 MB. Copyright © 2023 McAnulty et al. 2023 McAnulty et al. https://creativecommons.org/licenses/by/4.0/ This content is distributed under the terms of the Creative Commons Attribution 4.0 International license.

### Taxonomic diversity and variability of ANG communities from laboratory-raised animals.

While the bacterial community averages presented in the above sections provide some insight into the community composition of the ANGs of raised animals (Fig. 3A), the standard deviations associated with these averages (Fig. 3C to F) demonstrate the high variability of these taxa. For example, all raised ANG communities contained members of the *Rhodobacteraceae*, but in two of the CT-raised lab sand samples these OTUs accounted for less than 0.1% of the ANG community, while, in a third ANG from the same treatment, these OTUs accounted for 74% of the community (Fig. 3C). A scatterplot of the various bacterial families shows the high variability in the ANGs of raised versus wild-caught squid (Fig. 3C to F), both within and between conditions. Bray Curtis beta-diversity analysis revealed that CT-raised animals in lab sand and wet-collected sand resulted in a higher average (although not significant) distance to centroid (a measure of variability within a group), while the WI-raised lab sand and wet sand + *Leisingera* sp. JC1 conditions resulted in a lower average distance (Fig. 3B). While the lower variability from WI-raised animals may be unexpected, this finding can be explained by the highly homogenous community dominated by a single taxon, with one outlier that also contained more taxonomic variation, including a higher proportion of *Verrucomicrobia* (Fig. 3F).

### ANG bacterial composition of animals raised on wet-collected substrate had a community that most closely resembled that of wild-caught hosts.

**FIG 5:**
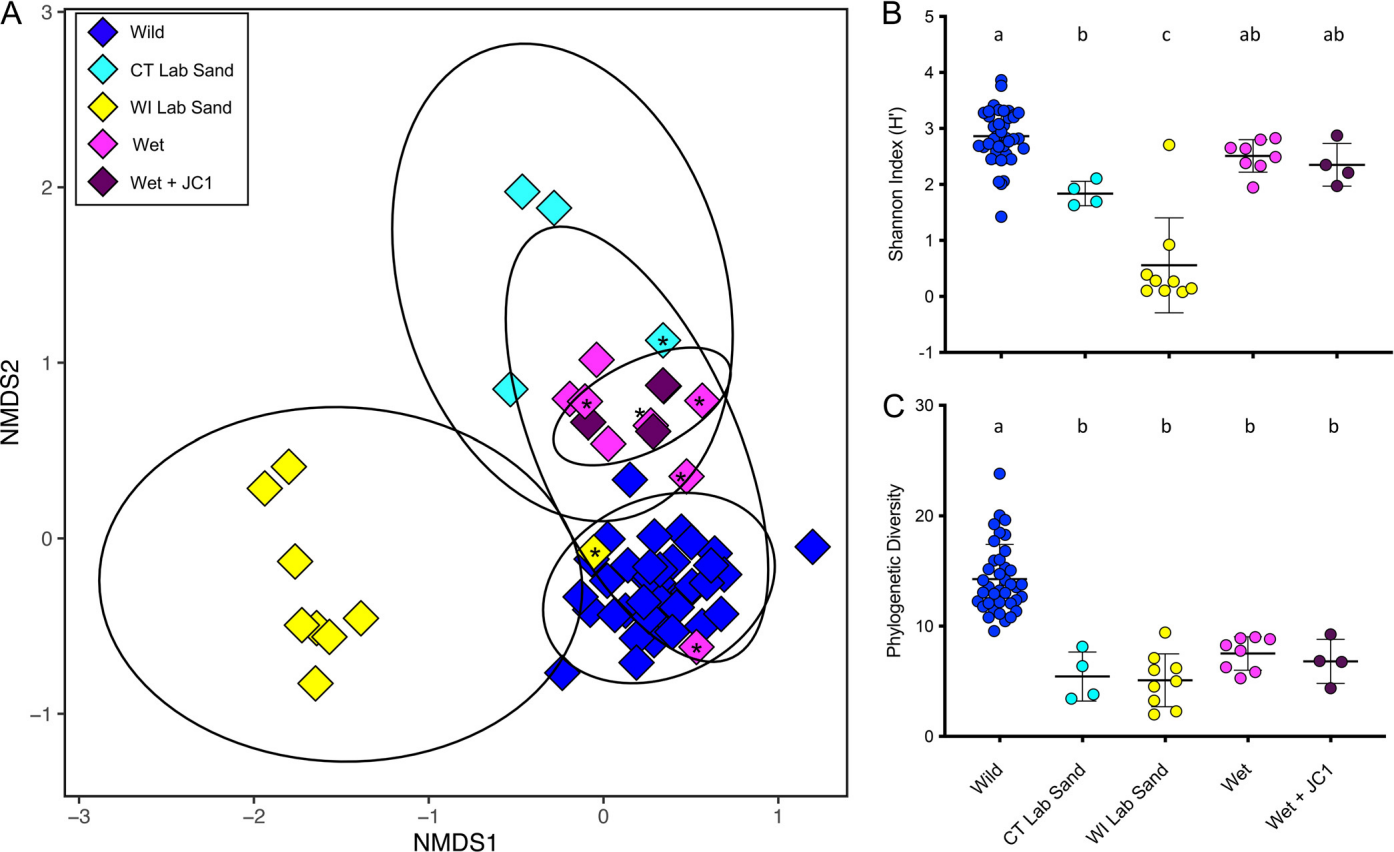
Bray Curtis beta diversity analysis demonstrates that the ANG bacterial composition of lab-raised animals was generally distinct from that of wild-caught *E. scolopes*, but those animals raised on wet-collected sand had a community that most closely resembled that of wild-caught animals (A). Asterisks indicate raised ANGs with >4% relative abundance of *Verrucomicrobia*, demonstrating that a higher abundance of *Verrucomicrobia* appears to shift the overall community composition toward that of the wild-caught animals (A). The richness and evenness of the community was not significantly lower than that of ANGs from wild-caught squid for animals raised on wet-collected sand (B), but the phylogenetic diversity of all ANGs from raised animals was significantly lower than that of wild-caught animals (C). Letter groups denote significantly different alpha diversity levels (B: F_4,58_ = 36.75, *P* < 0.0001; C: F_4,58_ = 32.34, *P* < 0.0001), based on one-way ANOVA and *post hoc* Tukey’s test for multiple comparisons. “Wet” indicates wet-collected sand throughout. “Lab sand” samples are from stunted ANGs from raising experiments not conducted on wet-collected sand (Fig. 2).

The alpha diversity of both CT- and WI-raised ANGs was lower than that of communities from wild-caught animals but varied by metric. All raising conditions where ANGs developed (Fig. 2) resulted in symbiotic communities with significantly lower phylogenetic diversity than those of wild-caught animals when examined by one-way ANOVA and *post hoc* Tukey’s tests (F_4,58_ = 32.34, *P* < 0.0001, Fig. 5C). The richness and evenness of the community as measured by the Shannon Index (H’) was significantly lower in the WI-raised animals than in any other raising conditions (F_4,58_ = 36.75, *P* < 0.0001, Fig. 5B). The CT-raised lab sand H’ was also significantly lower than wild-caught, although the wet-collected sand (with and without added *Leisingera* sp. JC1) H’ was not significantly different than the samples from wild-caught animals (F_4,58_ = 36.75, *P* < 0.0001, Fig. 5B). These results provide further evidence that substrate from the host’s natural environment provided the necessary cue for the formation of an ANG consortium that most resembles wild-caught hosts. Further support of this hypothesis comes from an examination of the Bray Curtis beta diversity analysis (Fig. 5A). WI-raised ANGs formed a tight cluster apart from ANGs from both the CT-raised and wild-caught squid. One WI-raised ANG clustered with the wild-caught squid ANGs. This ANG was the sole sample from its group that contained *Verrucomicrobia* at a substantive level (6.3%, Fig. 5A). Similarly, one CT-raised lab sand animal had an ANG community that clustered more closely to ANGs from wild-caught squid. This ANG was also the sole sample from that condition to contain *Verrucomicrobia* (8.0%, Fig. 5A). All the ANGs from animals raised on wet-collected sand (with and without added *Leisingera* sp. JC1) clustered more closely to ANGs from wild-caught animals than did any of the other raising conditions, again demonstrating the importance of substrate from the host’s natural habitat playing a role in the induction of ANG development.

### Changes of selected taxa within the substrate bacterial community of lab-raised animals.

**FIG 6:**
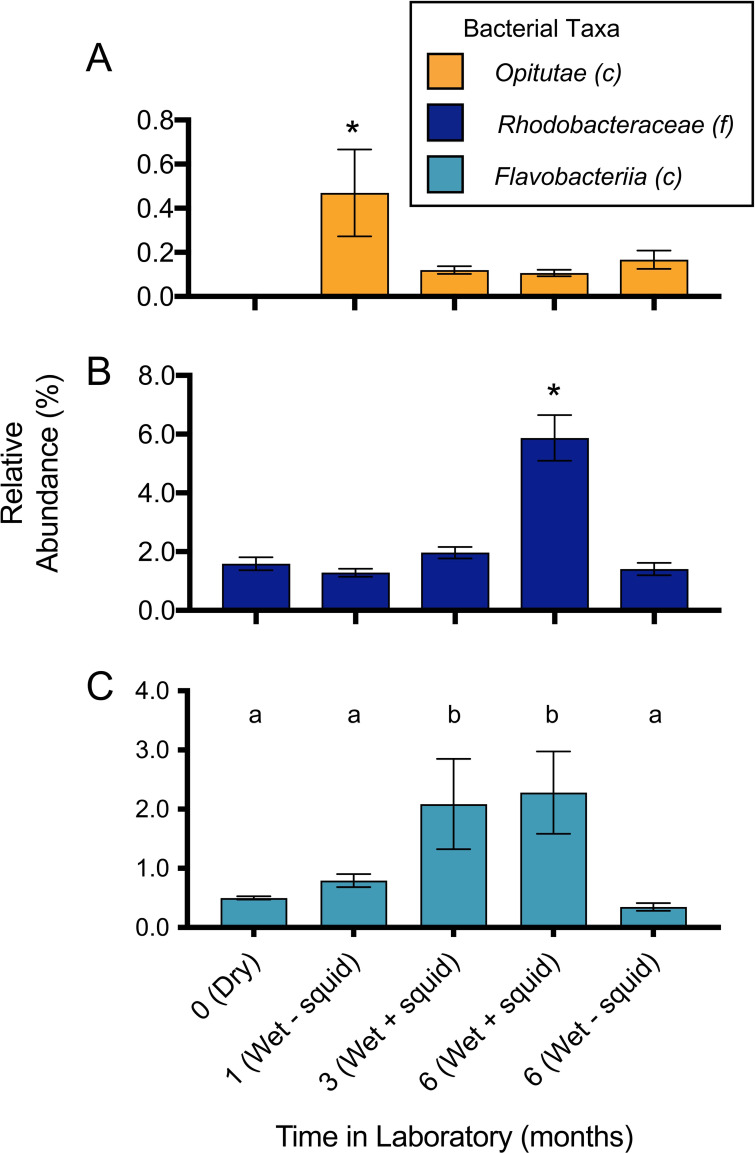
Changes of selected taxa within the sediment bacterial community of lab-raised animals. Sediment (sand) community was profiled (n = 3/time point) 1 month after arrival in lab, 3 months post collection (with squid present in tanks), and 6 months post collection (both with and without squid present in tanks). The community of dry-collected sand was also profiled when it first arrived in the lab. Asterisk denotes significantly different relative abundance levels (A: F_4,10_ = 11.34, *P* = 0.001; B: F_4,10_ = 75.65, *P* < 0.0001), as do letter groups (C: F_4,10_ = 11.49, *P* = 0.001), based on one-way ANOVA and *post hoc* Tukey’s test for multiple comparisons. *Opitutae* is a class of *Verrucomicrobia*; *Rhodobacteraceae* is a class of *Alphaproteobacteria*. Note differences in *y* axes for different taxa on bar graphs. “Wet” indicates wet-collected sand throughout. “Dry” indicates dry-collected sand throughout.

As ANGs from animals raised in CT with wet-collected Hawaiian sand were the only samples from laboratory-reared squid to contain a substantial proportion of *Verrucomicrobia* (on average 15.7 ± 7.3%, Fig. 3A), we examined the bacterial composition of the sand used for these raising experiments. Wet-collected sand from Maunalua Bay was analyzed 1 month post collection, 3 months post collection (tanks with squid), and then 6 months post collection (both from tanks with and without squid), and was compared to the community of sand taken directly from Maunalua Bay, HI (previously analyzed [33]) as well as dry-collected sand from Lanikai Beach, HI. This analysis showed that the microbial community in laboratory conditions was more constrained, and that it changed in composition over time, although less so when squid were absent from tanks (Fig. 6). While the taxa common between ANGs and the sediment community are present at low levels naturally (3.2% *Rhodobacteraceae* and 0.3% *Verrucomicrobia* (34)), we found that these taxa shifted significantly when sediment was maintained in the lab over a period of 6 months. The relative abundance of *Rhodobacteraceae* and *Flavobacteriia* increased significantly over that time, while the *Verrucomicrobia* decreased to almost undetectable levels within 3 months (F_4,10_ = 65.65, *P* < 0.0001 (*Rhodobacteraceae*); F_4,10_ = 11.34, *P* = 0.001 (*Verrucomicrobia*); F_4,10_ = 11.49, *P* = 0.001 (*Flavobacteriia*), Fig. 6). 10 of 21 OTUs found in at least 87.5% of ANGs from animals raised on wet-collected sand were also identified in the 1-month sand samples, representing 56.4% of the average ANG community (Table S3). The *Verrucomicrobia* OTUs found in ANGs were more variable but were also present in the 1-month sand community. The most abundant *Verrucomicrobia* OTU was present in 50% of the raised ANGs, and was also present in the initial sediment, averaging 0.18% of that community (Table S3). The dry-collected sand notably lacked *Verrucomicrobia* OTUs (Fig. 6A), while previous analysis of our laboratory substrate found that *Verrucomicrobia* was present in tanks with wild-caught squid at low abundances (0.004% [34]).

10.1128/mbio.02131-22.3 TABLE S3 Comparison of bacterial OTUs (operational taxonomic units) found in at least 87.5% of ANGs from animals raised on wet-collected sand (n = 8) and those present in wet-collected sand after one month in the lab. Download Table S3, TIF file, 1 MB. Copyright © 2023 McAnulty et al. 2023 McAnulty et al. https://creativecommons.org/licenses/by/4.0/ This content is distributed under the terms of the Creative Commons Attribution 4.0 International license.

## DISCUSSION

**FIG 7:**
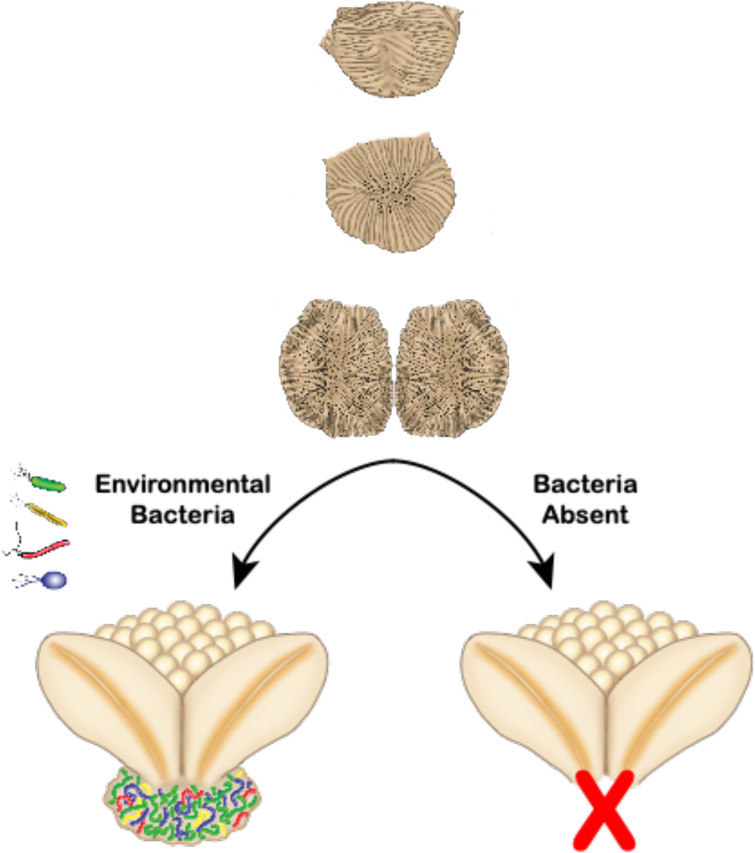
Model for lack of complete ANG development in the absence of environmental bacteria. The nascent ANG primordium forms over the first month after hatching and is poised to recruit environmental bacteria during colonization (40) (upper images). We hypothesize that if specific environmental bacteria are present during a colonization window, the adult ANG fully develops and is present at sexual maturity (lower left). If specific environmental bacteria (possibly *Verrucomicrobia*) are not present, the ANG primordium regresses, initiation of tubule development fails, and associated tissues do not form. The ANG is then absent in sexually mature adults that are raised in conditions with a reduced or inappropriate bacterial environmental background (lower right). Illustration by Virge Kask.

The results of this study suggest that bacteria from the environment of the Hawaiian bobtail squid are linked to the development of a symbiotic organ in *E. scolopes* (Fig. 7). Although there are many examples of the microbiota influencing the development of tissues, organs, and/or structures in animals and plants, we believe this is the first example to show that a symbiotic organ completely fails to form without exposure to symbiotic bacteria from the environment.

Bacteria can influence animal development in a number of ways. In the association between *E. scolopes* and V. fischeri, the symbiont is involved with triggering morphogenesis of the nascent light organ, although the light organ accessory tissues will still develop in the absence of the symbiont (8). Some organ systems remain underdeveloped in the absence of a bacterial signal. For example, in gnotobiotic mice, the capillaries of the gut-associated lymphoid tissue remain stunted unless a conventional microbiome is present (6). In addition to decreased vasculature development, germfree mice exhibit abnormal immune systems, including structural differences, and fewer immune cells and effectors (42, 43). In multiple beetle species, bacterial endosymbionts are responsible for formation and hardening of the beetle’s cuticle (44, 45). The molecular signaling between the host-microbe partners that leads to proper organ development is frequently uncharacterized, but microbe-associated molecular patterns (MAMPs) have been implicated in some cases (7, 46–49). However, with the exception of root nodules in leguminous plants (19), organogenesis still occurs in the absence of the conventional environmental microbiota, although organs may fail to develop properly. For example, in mice, intestinal microbiota influence the development of specific lineages of lymphocytes (T-cells), but T-cells and the thymus are still present (50).

The process of mammalian organogenesis has been assumed to occur largely independently of symbiosis; however, in some animals and plants, bacteria are known to influence reproduction and/or organ/tissue development. In the woodlouse, *Armadillidium vulgare*, the presence of the bacterium *Wolbachia* prevents the formation of the androgenic gland, leading to feminization of the host (51). In the planarian, *Dugesia japonica*, a bacterial indole from *Aquitalea* sp. influences head patterning during regeneration (52). In the plant Arabidopsis thaliana the presence of the rhizobacterium Phyllobacterium brassicacearum results in a delay in flowering, and subsequently in a larger reproductive biomass in the adult plant (53). Here, we show that female bobtail squid raised in the absence of natural environmental microbiota often failed to develop the ANG. A few of these animals had severely stunted organs and divergent microbial communities (Fig. 1–3, 5) but in all of these cases the animals had been transferred to adult tanks that harbored environmental and/or ANG bacteria from wild-caught animals. Furthermore, the development of the ANG was independent of light organ colonization by V. fischeri (Table S2), and the addition of some cultured members of the ANG consortium was not sufficient to induce complete organ development (Fig. 2). These data suggest that development of the mature ANG does not occur in response to the general presence of bacteria, regardless of concentration, particularly since the gland is in close proximity to the light organ, which expels high cell densities of V. fischeri daily (54). While the substrate from WI-raised animals was not sampled for 16S rRNA gene community sequencing, the consistent development of ANGs in CT-raised animals that were raised under conditions where sand from the host’s natural environment was also present supports our conclusions. ANGs from WI-raised animals appeared smaller than those from wild-caught squid and had a modified bacterial community, while hosts raised entirely on unaltered sand from the host’s natural environment developed ANGs that most resembled those from wild-caught adults (both morphologically and in bacterial composition; Fig. 1, 3, 5). Additionally, we were able to introduce a cultured isolate into the ANG when squid were raised with wet-collected natural environmental sand (Fig. 4). Overall, these data suggest that bacteria from the bobtail squid’s environment induce development of the ANG.

Our data also support a model of environmental transmission for the ANG symbiosis. Juvenile *E. scolopes* do not develop a visibly detectable ANG until they reach approximately 10 mm mantle length,1 to 1.5 months posthatching (40, 55). Previous studies have found that in the market squid, *Doryteuthis pealeii*, the closest relatives to ANG symbiont strains were environmental (56), and that in the inshore squid, *Doryteuthis opalescens*, the veined squid, *Loligo forbesii*, and the elegant cuttlefish, *Sepia elegans*, colonization of the ANG is an extended process, happening over weeks of development (57–59). In *E. scolopes* the nascent ANG forms first as an epithelial field which then develops extensive pores connected to ciliated invaginations over the course of several weeks (40). In the ANGs of these decapod cephalopods, nascent tissues appear poised to recruit bacteria from the environment. In *E. scolopes*, these recruitment tissues regress by the time the squid reach maturity (40). Consistently, in this study, no sign of a persistent nascent organ or primordium was observed in adult animals that failed to develop ANG tubules. We hypothesize that the nascent ANG develops regardless of a bacterial “signal” but fails to mature into a fully formed adult ANG unless exposed to proper environmental bacteria during a colonization window (Fig. 7). Previous work examining bacterial composition of the seawater and sediment in the natural environment of the Hawaiian bobtail squid found that a majority of the OTUs present in the symbiosis are also present in the environment (34). The ANG community of wild-caught squid was unaffected when those squid were transferred to and maintained in the lab for up to 4 months (33, 34), indicating that ANG tubules were initially colonized and then maintained throughout the life of the host, similar to the light organ symbiosis (60). The ANG community members of *E. scolopes* appear to colonize the host early in development, although shifts in the relative abundance of those members continue throughout development of the ANG until maturity is reached (40). Here, we demonstrate that juvenile squid raised in the laboratory from hatching do not have the same ANG bacterial composition as wild-caught animals unless bacteria from the host’s natural environment are present. We also provide evidence that specific bacterial strains can be incorporated into the ANG community once full organ development is induced. Together, these data provide strong evidence for environmental transmission of the ANG symbiosis that is linked to development and maturation of the gland.

In the light organ of *E. scolopes*, colonization by V. fischeri causes rapid morphogenesis and the regression of recruitment tissues, the ciliated epithelial appendages (23). Regression of the ANG recruitment tissues does not occur as rapidly after colonization, and also takes place over a longer duration (40). The consortial nature of this symbiosis may indicate that more than a single type of bacteria is required to induce full, normal development of the organ. This hypothesis may explain the presence of a few asymmetrical and stunted ANGs observed in CT-raised squid that were exposed to laboratory sand containing some bacteria from the host’s natural environment, as well as their lower alpha diversity and dominance by *Alphaproteobacteria*, which are more often present under laboratory conditions (Fig. 3, 5, 633). Characterizing the early onset of the ANG symbiosis with more cultured strains (i.e., from the *Verrucomicrobia*) should help demonstrate the mechanisms by which microbiota induce development of the ANG.

The particular bacterial species from *E. scolopes’* natural environment that are responsible for initiating ANG development are currently unknown, but our collective data suggest that *Verrucomicrobia* may play a role. The bacterial composition of CT-raised ANGs from wet-collected sand was most similar to the community found in ANGs of wild-caught squid. Our analysis of the bacterial diversity from the wet-collected sand demonstrates that the bacterial community changes dramatically over time in the laboratory. The relative abundance of *Verrucomicrobia* in wet-collected sand rapidly declined to almost undetectable levels over a period of months and was not detected in the dry-collected sand that had a depleted microbial community and failed to initiate development of ANGs (Fig. 6A). The shift in the bacterial composition of the sand kept in the laboratory over time reflects the overall shift in the bacterial community of raised animals compared to that of wild-caught squid. Low levels of *Verrucomicrobia* have previously been detected in the substrate of tanks containing wild-caught squid (34), possibly explaining the formation of stunted ANGs from squid transferred to these tanks midway through development. Verrucomicrobia was also detected in higher abundance in ANGs from *E. scolopes* with smaller mantle lengths, suggesting that the ANG community shifts as squid mature and reach sexual maturity (40). A similar shift has been suggested in the symbiotic community of the bigfin reef squid, *Sepioteuthis lessoniana* during ANG development (61). *Verrucomicrobia* from the ANGs of *E. scolopes* have not yet been cultured, but efforts are under way using metagenomics and transcriptomics to understand the metabolic requirements of this group. Determining the specific bacteria necessary to induce full organ development and the mechanisms behind this induction will be important avenues of future research.

Other possible inducers of ANG development may include Archaea, Eukarya, and nonliving factors (i.e., secreted peptides, temperature, etc.). Archaea were not detected in the developing ANG via 16S rRNA amplicon sequencing (40), nor were eukaryotic cells detected via confocal and transmission electron microscopy imaging (40). Our experiments included an autoclaved sand control (wet-collected sand, Fig. 2) which would have included the same non-living components as non-autoclaved, wet-collected Hawaiian sand, but which did not induce ANG development. These results suggest that nonliving factors are unlikely to trigger ANG development although we do not rule out a role for microbial products that may have been inactivated or denatured by autoclaving (e.g., MAMPs such as lipopolysaccharide (LPS), peptidoglycan, etc.). Together, these results suggest that bacteria from the host’s natural environment induce ANG development. While the mechanism behind ANG organogenesis is not yet understood, the results presented here provide an important advance in determining the specific bacteria and signals that lead to full ANG development.

The Hawaiian bobtail squid has served as a valuable symbiosis model for understanding the light organ association with V. fischeri (31, 32). Raising squid in the presence or absence of V. fischeri has allowed for powerful genetic and morphological comparisons that have revealed mechanisms of how beneficial bacteria influence animal development (31, 32). Much focus in this system has been on how MAMPs from V. fischeri such as LPS, peptidoglycan, and tracheal cytotoxin (TCT) influence light organ morphogenesis (31, 32, 49) and our future research with the ANG association will explore the role that MAMPs may play in development of the gland. The ANG association is also an emerging model system for studying defensive consortial symbioses (31, 62). The ability to manipulate the development of the ANG symbiosis has, until now, not been feasible in laboratory culture, but the advancements described in this paper have elevated the tractability of this system, allowing for finer control of the ANG symbiosis for experimentation going forward. Our results demonstrate that ANG developmental outcomes can be predicted under laboratory conditions. The ability to experimentally manipulate both a binary (light organ) and a consortial (ANG) symbiosis within the same host demonstrates the experimental power of the Hawaiian bobtail squid as a model organism for symbiosis research. The results presented here also reveal the potential to develop gnotobiotic squid for both organ associations. Gnotobiotic squid with defined ANG communities, along with gene expression analyses, would allow for investigation into the specific host-microbe interactions which lead to tubule formation and complete ANG development, while also permitting manipulation of the community present in squid eggs to further understand the functional role of this symbiosis. The bacterial consortium present in the eggs can prevent fungal fouling, and individual isolates can inhibit both fungi and bacteria (35, 38, 39). In the future we will investigate whether eggs from females that fail to develop an ANG are more susceptible to fouling and/or pathogenic microbes. Whole-genome sequencing of *E. scolopes* revealed that the light organ and ANG likely evolved by different mechanisms (63), with the ANG displaying an enrichment of expressed orphan genes unique to *E. scolopes*. The role of orphan genes in the evolution of novel structures in animals has been described in a number of other systems (e.g., cnidocytes in cnidarians [64]) and the ability to manipulate ANG development will allow for a greater understanding of how interactions between environmental bacteria and animal hosts lead to organ development.

## MATERIALS AND METHODS

### Animal collection and general raising.

All animal experiments were conducted in compliance with protocol number A18-029 approved by the Institutional Animal Care and Use Committee, University of Connecticut. Adult Hawaiian bobtail squid were collected from Maunalua Bay, Oahu, Hawaii (21°16’51.42″N, 157°43’33.07″W), and were transported to either the University of Connecticut or the University of Wisconsin. Bobtail squid were maintained in aerated aquaria with circulating water and fed a diet of freshwater or marine glass shrimp. Adult animals were mated periodically, and the resulting eggs were collected and transferred to a nursery for the duration of embryogenesis (~3 weeks). Hatchling bobtail squid were moved to modified horse feed trough tanks (Fortiflex, Durado, Puerto Rico), and were raised under several different conditions (described below). In Wisconsin, squid (n = 50) were raised as previously described (60) with sand that was originally imported from Oahu but which had been in the lab for several months to years.

In Connecticut, raising proceeded with three distinct methods (Fig. 2). In all cases bobtail squid were fed live mysid shrimp until they were large enough to hunt for small glass shrimp, at which point they were transferred to a live glass shrimp diet.

**(i) CT-raised lab sand conditions.** Hatchling bobtail squid were raised in 0.22 μm filter-sterilized artificial seawater (FSSW, Instant Ocean, Blacksburg, VA) on autoclaved quartz sand. Although the initial sand and water were sterile, the necessary addition of live food during raising ensured that raising conditions were not sterile, although laboratory seawater and sand bacterial communities typically found in our system were depleted or absent. For these initial experiments and in order to better keep track of individual animals, squid under this condition were transferred to tanks at approximately 10 mm mantle length in which wild-caught adult squid had previously been maintained and for which the sand and circulating FSSW had not been sterilized. Size of lab-raised squid has previously been found to loosely correlate with age and developmental stage of the ANG (40). Sand in these tanks was originally collected from Hawaii, approximately 1 year before the onset of the experiments. While we are using the term “sand” throughout the manuscript, the sediment includes a mix of sand and silt.

**(ii) Wet-collected condition.** Wet Maunalua Bay sand was collected from below the low tide line and shipped to Connecticut. Upon arrival, sand was immediately transferred to aerated troughs. Bobtail squid were raised in unfiltered artificial seawater on this wet-collected sand from hatching to adulthood. Upon dissection, ANGs were imaged using a Zeiss Discovery v20 Stereo scope.

In separate trials, animals were raised on wet-collected sand with the addition of *Leisingera* sp. JC1 (38), which was added to artificial seawater daily at 10,000 CFU/mL throughout the raising period (from 10 days of age until a mantle length of 20 mm was reached). A high concentration of *Leisingera* sp. JC1 was added in this experiment because we wanted to increase the availability of symbionts for colonization. This natural strain was originally isolated from wild-caught bobtail squid eggs from Maunalua Bay (38). Squid were sacrificed after reaching 20 mm mantle length and the ANG was surface sterilized by washing in 99% ethanol and filter-sterilized squid Ringer’s solution (FSSR [34]). ANGs were then homogenized in FSSR and serial dilutions were plated in triplicate onto seawater tryptone agar plates (65). Plates were incubated overnight at 28°C and examined for the characteristic blue-black colonies formed by *Leisingera* sp. JC1 (38). Those colonies were counted to determine the CFU/ANG of this environmentally available strain. To support that blue-black colonies were *Leisingera* sp. JC1, colony PCR was performed on 3 blue-black colonies from each ANG to confirm presence of the pigment (indigoidine) biosynthesis genes, *igiCDR* (Table S4). The RNA polymerase beta subunit gene, *rpoB*, was used as a positive control. PCR was performed with GoTaq polymerase, for 36 cycles at an annealing temperature of 55°C.

10.1128/mbio.02131-22.4 TABLE S4 Primer sequences used in this study. Download Table S4, TIF file, 1 MB. Copyright © 2023 McAnulty et al. 2023 McAnulty et al. https://creativecommons.org/licenses/by/4.0/ This content is distributed under the terms of the Creative Commons Attribution 4.0 International license.

**(iii) Dry-collected condition.** Dry Hawaiian sand was collected from Lanikai Beach, Oahu, Hawaii (21°23'33.3″N, 157°42'54.6″W). This sand was chosen for its fine grain, which allows small squid to bury easily. Lanikai sand was collected from a dry area of the beach above the high-tide line. Sand was strained to remove debris and washed thoroughly with DI water after being shipped to CT. Bobtail squid were raised on this sand from hatching to adulthood.

Two additional conditions were performed on dry-collected sand. For the first, three strains of *Rhodobacteraceae* (*Alphaproteobacteria*), *Leisingera* spp. JC1 and ANG1, and *Tateyamaria* sp. S1, were added to tank water daily at 1,000 CFU/mL (concentration of each strain individually). These strains were originally isolated from the ANG or egg jelly coat communities [Table S1, (33, 37, 38)]. These strains were chosen because they are all members of the *Alphaproteobacteria*, which is the most abundant bacterial class in 16S rRNA community profiling of the ANG (33, 34, 36). We inoculated squid at 1000 CFU/mL based on the knowledge that this concentration is sufficient for light organ colonization by V. fischeri (23). For the second condition, a more phylogenetically diverse set of three ANG strains was added to the tank water daily: *Leisingera* sp. ANG-DT (*Alphaproteobacteria*), *Vibrio* sp. ANG51 (*Gammaproteobacteria*), and *Muricauda* sp. ANG21 (*Flavobacteriia*) at 10,000 CFU/mL (concentration of each strain individually, Table S1). We hypothesized that a more diverse bacterial community could lead to successful colonization of the ANG, and each of these strains was from a different bacterial class (*Alphaproteobacteria*, *Gammaproteobacteria*, and *Flavobacteriia,* respectively). The concentration of bacteria was increased to 10,000 CFU/mL to increase availability of cells for colonization compared to the previous treatment.

### DNA extraction, amplification, sequencing, and analysis.

Raised bobtail squid were sacrificed once fully mature (mantle length > 20 mm) and dissected to remove the ANG. ANG bacterial DNA extraction was completed as previously described (34). Briefly, tissues were surface sterilized by washing in 99% ethanol and FSSR. ANGs were then homogenized in FSSR with a plastic pellet pestle (Thermo Fisher Scientific, Waltham, MA) and differentially centrifuged to separate bacterial cells from host cells (homogenized ANGs were centrifuged at 100 × *g* for five min to pellet the host tissue, the supernatant containing the bacterial cells was then removed and centrifuged at 5,000 × *g* to pellet the bacteria). DNA was extracted from the bacterial fraction using bead beating with zirconia beads (0.1 and 0.5 mm) for 3 min, and the DNeasy blood and tissue kit (Qiagen, Valencia, CA). DNA concentration was determined using the Qubit dsDNA High Sensitivity assay (ThermoScientific, Waltham, MA) and averaged 37.2 ± 21.3 ng/μL (all samples > 3.0 ng/μL).

Sediment samples were collected in triplicate at four time points over the course of experiments. Wet-collected sand was sampled 1 month after arrival in the lab from Hawaii, after 3 months in a tank with developing bobtail squid, and after 6 months in a tank with developing squid. Wet-collected sand was also sampled after 6 months from a storage tank where no squid were housed. Dry-collected sand was sampled immediately upon arrival in the lab. DNA extraction was completed as previously described (34) using the DNeasy blood and tissue kit with bead beating. DNA concentrations averaged 32.2 ± 10.8 ng/μL (all samples > 18 ng/μL).

The V4 region of the 16S rRNA gene was amplified from ANG and sediment extractions using barcoded primers developed by Caporaso et al. (66) and sequenced on an Illumina MiSeq sequencer (Illumina, San Diego, CA) according to established protocols (34, 67). Samples were processed either in the Nyholm lab or at the University of Connecticut Microbial Analysis, Resources and Services facility (MARS). Averages were obtained for CT-raised lab sand ANG samples of 126,741 ± 56,288 reads/sample (n = 4, minimum 72,077 reads/sample), 38,158 ± 12,374 reads/sample for wet-collected sand ANG samples (n = 8, minimum 24,210 reads/sample), 35,203 ± 14,051 reads/sample for wet-collected sand + JC1 ANG samples (n = 4, minimum 21,605 reads/sample) and 52,996 ± 13,873 reads/sample for WI-raised ANG samples (n = 9, minimum 33,065 reads/sample). An average of 29,840 ± 7,752 reads/sample was obtained for sediment samples (n = 12, minimum 20,949 reads/sample). ANGs from wild-caught squid were previously published and reanalyzed for this study and contained an average of 74,739 ± 31,370 reads/sample for ANGs collected in Maunalua Bay (34), 50,052 ± 12,197 reads/sample for ANG samples collected in Kaneohe Bay (36), and 18,753 ± 7,103 reads/sample for sediment (34).

Negative-extraction (no-sample) and PCR (no-template) controls were processed and sequenced simultaneously with all samples and yielded less than 1,000 sequences/control in all cases. The majority of sequences in these controls were associated with a single Escherichia OTU, and most of the other sequences were associated with non-ANG OTUs. Control samples did contain three *Rhodobacteraceae* OTUs associated with the ANG community but accounted for less than 1% of sequences within the control samples. The presence of *Rhodobacteraceae* in the ANG was previously established through the use of fluorescence *in situ* hybridization (33) and culturing techniques (37). No *Verrucomicrobia* OTUs were found in any of the control samples.

QIIME1 was used to analyze sequencing data (68) following established protocols (36, 67). Briefly, operational taxonomic units (OTUs) were assigned with *de novo* methods at the 97% identity level. All samples were rarified to 15,000 sequences. Alpha diversity and beta diversity were analyzed using QIIME1, Prism, and the R vegan package. Statistical analyses of alpha diversity variation and beta diversity distance to centroid were completed in Prism as one-way ANOVAs followed by *post hoc* Tukey’s tests.

### Data availability.

Sequence data were compared to ANG community data previously published under the project ID ENA PRJEB14655, accession numbers ERS1496678-ERS1496701, ERS1496705-ERS1496706, and ERS1498392-ERS1498393 (34) and under the project ID ENA PRJEB23264, accession numbers ERS1994108-ERS1994112, and ERS2012872-ERS2012876 (36). New sequence data were deposited in the European Nucleotide Archive (ENA) under the project ID PRJEB30950. All previously published ANG data were reanalyzed for this study.
